# Ethnic–Racial and Spatial Inequalities of American Cutaneous Leishmaniasis in Brazil: A Nationwide Analysis of Cumulative Notification and Mortality

**DOI:** 10.3390/pathogens15070719

**Published:** 2026-07-08

**Authors:** Alís Hassan Salman, Lara Nazareth Afonso, Ana Jullia Alves Guimarães, Andressa Alves Soares, Paula Pereira da Matta, Ari Sérgio de Oliveira Lemos, Patrícia de Almeida Machado, Juliana da Trindade Granato

**Affiliations:** 1 IDOMED—Instituto de Educação Médica, Angra dos Reis 23914-360, Brazil; salmans6@hotmail.com (A.H.S.); anajullia.ja@gmail.com (A.J.A.G.); andressaalves9772@gmail.com (A.A.S.); paulap.damatta11@gmail.com (P.P.d.M.); 2 Departamento de Patologia, Faculdade de Medicina, Universidade Federal Fluminense (UFF), Niterói 24220-900, Brazil; laranazareth@id.uff.br (L.N.A.); patricia_machado@id.uff.br (P.d.A.M.); 3 Núcleo de Pesquisa em Parasitologia, Universidade Federal de juiz de Fora (UFJF), Juiz de Fora 36036-900, Brazil; ariufjf@gmail.com

**Keywords:** american cutaneous leishmaniasis, ethnic-racial inequalities, spatial analysis

## Abstract

The Pan American Health Organization (PAHO) recognizes ethnicity as a structural determinant of health, yet ethnic–racial analyses of neglected tropical diseases remain scarce. This study investigated cumulative notification and mortality rates of American cutaneous leishmaniasis (ACL) in Brazil from 2016 to 2025 according to the official Brazilian race/color categories. Data were obtained from the Notifiable Diseases Information System (SINAN/DATASUS), and temporal and spatial patterns were analyzed. ACL showed marked geographic heterogeneity, with the highest cumulative notification rates concentrated in the North and Central-West regions. Indigenous and Asian populations presented the highest cumulative notification rates, whereas mortality rates were highest among Indigenous populations and remained elevated among Brown populations in several settings. Spatial clusters were concentrated mainly in the Brazilian Amazon, reinforcing the persistence of territorial inequalities in ACL distribution. Overall, the findings reveal substantial race/color and geographic disparities in both ACL occurrence and mortality in Brazil, underscoring the need for more equitable surveillance, prevention, and control strategies.

## 1. Introduction

Leishmaniasis is classified by the World Health Organization (WHO) as a Neglected Tropical Disease and is caused by protozoa of the genus *Leishmania* [1,2]. Transmission among vertebrate hosts occurs through the bite of infected female phlebotomine sand flies during blood feeding [3]. To date, at least 21 *Leishmania* species capable of causing human infection have been described in the literature [4]. It is important to emphasize that leishmaniases constitute a complex group of diseases characterized by considerable epidemiological diversity and a broad global distribution, affecting more than 90 countries and territories. Approximately one billion people live in areas at risk of infection worldwide [3,5].

In Brazil, American Cutaneous Leishmaniasis (ACL) represents a major public health concern. It is therefore classified as a notifiable disease, requiring mandatory reporting through the National System for Notifiable Diseases (SINAN) [6]. Over the past decade, 224,311 ACL cases have been reported nationwide, with the states of Pará, Bahia, Mato Grosso, Maranhão, Amazonas, and Minas Gerais accounting for more than 67% of all notifications [7]. The epidemiology of ACL in Brazil is strongly influenced by territorial and socioeconomic characteristics, including agricultural activities, environmental modifications, and disparities in access to healthcare services. Together, these factors contribute to marked spatial heterogeneity in disease occurrence and burden, with higher concentrations of cases observed in regions undergoing intense environmental transformation, as well as in rural and forested areas where access to healthcare remains limited [8].

Brazil, which had a population of 203.08 million inhabitants in 2022, is marked by substantial racial diversity. According to the national census, 45.3% of the population self-identified as Brown (*pardo*), 43.5% as White (*branco*), 10.2% as Black (*preto*), 0.4% as Asian (*amarelo*), and 0.6% as Indigenous (*indígena*) [9]. This diversity coexists with persistent social inequalities, reflected by a Gini coefficient of 0.506 in 2024 and strongly shaped by regional disparities [10]. These inequalities disproportionately affect Afro-descendant and Indigenous populations, many of whom experience inadequate housing conditions and limited access to essential services, including sanitation and safe drinking water. Such structural vulnerabilities increase exposure to infectious diseases and their vectors, thereby exacerbating health inequities [11,12].

Recognizing these challenges, the Pan American Sanitary Conference and the Pan American Health Organization (PAHO) have urged member states to acknowledge ethnicity as a structural determinant of health and to develop evidence-based public policies aimed at reducing historically rooted inequalities [13]. Nevertheless, despite increasing recognition of the ethnic–racial dimensions of health disparities, epidemiological studies examining neglected tropical diseases through this lens remain limited.

In this context, investigations addressing the distribution of ACL among different ethnic–racial groups are essential for understanding how social, historical, and territorial factors shape patterns of vulnerability in endemic settings. By using Brazil as a case study, this research contributes to the international discussion on health inequities by generating evidence that may be applicable to other countries characterized by ethnic diversity, social inequality, and endemic neglected tropical diseases. Such evidence is fundamental for informing surveillance, prevention, and control strategies that incorporate principles of health equity and social justice.

Accordingly, this study aimed to estimate and analyze the cumulative notification and mortality rates of ACL across Brazil, stratified by ethnic–racial groups (Asian, White, Indigenous, Brown, and Black populations). In addition, we sought to characterize the temporal trends and spatial distribution of these indicators at both state and macroregional levels, while evaluating the potential influence of incomplete race/color information on the observed patterns. By integrating epidemiological, ethnic–racial, and territorial dimensions, this study seeks to identify disparities in disease burden and outcomes, thereby contributing to a more comprehensive understanding of ACL-related vulnerabilities in Brazil.

## 2. Materials and Methods

### 2.1. Study Area

Brazil, the largest country in Latin America, has an estimated population of approximately 203 million inhabitants distributed across 27 federative units and five macro-regions: North, Northeast, Central-West, Southeast, and South. The country exhibits considerable demographic, socioeconomic, and environmental diversity, shaped by distinct historical trajectories of territorial occupation, urbanization, and economic development [9].

Significant regional disparities are observed throughout the national territory. While the Southeast is the most populous region and concentrates a substantial share of the country’s economic activity, the North encompasses the largest territorial area and includes extensive forested and sparsely populated zones. These contrasts influence living conditions, access to healthcare services, and environmental exposures, making Brazil a particularly relevant setting for the investigation of spatial patterns and health inequalities [10].

### 2.2. Study Design and Data Sources

This cross-sectional study was based on secondary data obtained from official Brazilian health information systems. Data on reported cases of American Cutaneous Leishmaniasis (ACL) recorded between 2016 and 2025 were extracted from the Notifiable Diseases Information System (SINAN), available through the DATASUS platform, in May 2026, after the end of the 2025 surveillance year. Data on ACL-related deaths were also obtained from SINAN using the case outcome variable available in the notification records. Cases were analyzed according to race/color categories and geographic units throughout Brazil.

Population denominators were obtained from the 2022 Demographic Census conducted by the Brazilian Institute of Geography and Statistics (IBGE), the most recent national census available. These data were used to calculate cumulative ACL notification rates and mortality rates according to race/color and geographic region.

Race/color information was classified according to the official IBGE framework and based on self-identification. The following categories were considered: White (*branco*), Black (*preto*), Brown (*pardo*), Asian (*amarelo*), and Indigenous (*indígena*). The use of these categories enabled the assessment of ethnic–racial inequalities in ACL notification and mortality patterns across Brazil.

### 2.3. Cumulative ACL Notification Rates

Cumulative ACL notification rates were calculated for the overall population and stratified by race/color using the same standardized formula (Equation (1)). For the overall estimate, the numerator corresponded to all ACL cases reported to SINAN between 2016 and 2025, whereas the denominator corresponded to the population estimate obtained from the 2022 Brazilian Demographic Census (IBGE). For race/color-specific estimates, the numerator included only cases reported within each race/color category, and the denominator corresponded to the respective population estimate for that category. Population estimates from the 2022 Brazilian Demographic Census were used because they represent the most recent official population counts available for all ethnic–racial groups in Brazil. Notification rates were expressed per 1000 inhabitants to allow comparisons among states and ethnic–racial groups with different population sizes. To assess the potential impact of incomplete race/color information on the estimates, the proportion of records classified as “unknown” race/color was calculated (Equation (2)). This indicator was defined as the number of notifications recorded as “unknown” (“ignorado” in the original SINAN database) divided by the total number of reported cases, multiplied by 100.

(1) $$Cumulative\;rate=\left(\frac{N\;cases}{N\;populations}\right)\times 1000$$

(2) $$\%Unknown=\left(\frac{N\;unknown}{N\;total\;reported\;cases}\right)\times 100$$

### 2.4. ACL Mortality Rates

ACL mortality rates were calculated for the overall population and stratified by race/color and health region. Deaths were identified using the outcome variable available in the SINAN database, considering only notifications classified as “death due to ACL” (“Óbito por LTA”). Notifications classified as “death from other causes” were excluded from the analyses. Mortality rates were calculated according to Equation (3), using population estimates from the 2022 Brazilian Demographic Census (IBGE) as denominators. For race/color-specific analyses, only records with available race/color information were included. Mortality rates were expressed per 100,000 inhabitants. The mortality estimates presented in this study were derived exclusively from SINAN records and represent deaths attributed to ACL according to the outcome registered in the notification system.

(3) $$Mortality\;rate=\left(\frac{N\;deaths\;due\;ACL}{N\;populations}\right)\times 100.000$$

### 2.5. Spatial Analysis and Choropleth Map Construction

A georeferenced database was created for all Brazilian states, containing population data and the corresponding cumulative ACL notification and mortality rates for the overall population and for each official race/color category. These epidemiological indicators were linked to the Brazilian state shapefile and processed in QGIS software (version 3.34.13; QGIS Development Team, QGIS Association) for spatial visualization and map construction. Official cartographic layers obtained from the Brazilian Institute of Geography and Statistics (IBGE) were used as the geographic base to define state boundaries and support the spatial representation of cumulative notification and mortality rates across the country. Choropleth maps were generated separately for the overall population and for each race/color group using graduated color classes to represent the magnitude of the indicators in each state. For cumulative ACL notification rates, six fixed class intervals were defined and applied uniformly across all maps to allow direct visual comparison between the overall population and race/color groups. Mortality maps were also constructed using six graduated classes, with intervals defined according to the distribution and range of observed mortality values. The resulting choropleth maps were used as exploratory tools to identify geographic patterns and areas with higher cumulative ACL notification and mortality rates, supporting the interpretation of spatial inequalities in the distribution of ACL in Brazil.

### 2.6. Statistical Analysis

Cumulative ACL notification rates and mortality rates were calculated according to the formulas described in Section 2.3 and Section 2.4. Corresponding 95% confidence intervals (95% CI) were estimated using exact Poisson methods in Stata version 19.1 (StataCorp, College Station, TX, USA). Pearson’s correlation analysis was performed to evaluate the relationship between cumulative ACL notification rates and mortality rates across Brazilian states. Correlation coefficients (r) and corresponding *p*-values were calculated using GraphPad Prism software (version 8.0.2, San Diego, CA, USA), which was also used to generate graphical representations of notification and mortality data, including temporal trend graphs, bar charts, and comparative visualizations. Statistical significance was defined as *p* < 0.05. Temporal trends in ACL notification rates were assessed using the Joinpoint Regression Program (National Cancer Institute, Bethesda, MD, USA). Annual Percent Change (APC) and corresponding 95% confidence intervals were estimated to identify significant changes over the study period. Spatial analyses were conducted using QGIS software (version 3.34.13; QGIS Development Team, QGIS Association). Choropleth maps were generated to visualize the geographic distribution of cumulative ACL notification and mortality rates across Brazil. Local Moran’s I (Local Indicators of Spatial Association—LISA) was applied to identify statistically significant spatial clusters and spatial outliers. Spatial patterns were classified as High–High (HH), Low–Low (LL), High–Low (HL), or Low–High (LH), adopting a significance threshold of *p* < 0.05.

## 3. Results

### 3.1. Temporal and Spatial Distribution of ACL

**Figure 1:**
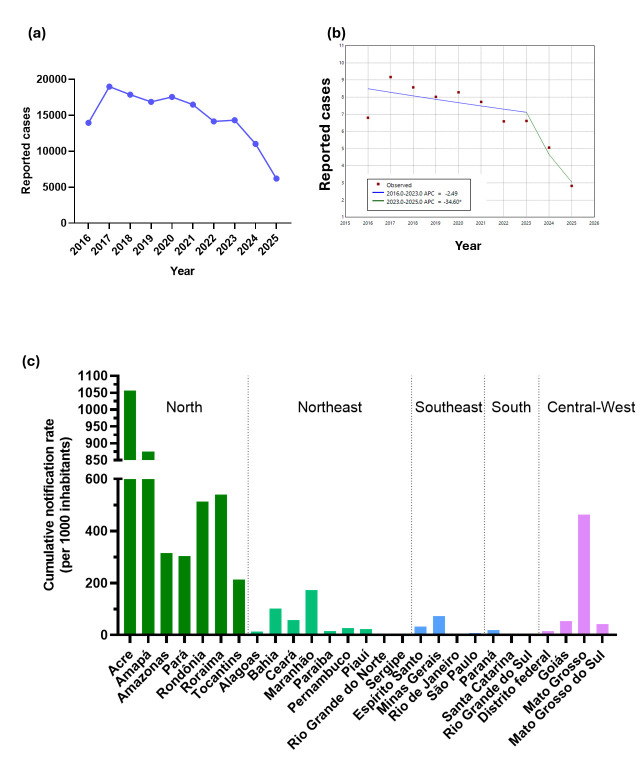
Temporal trends and geographic distribution of ACL in Brazil from 2016 to 2025. (**a**) Annual number of reported ACL cases nationwide. (**b**) Joinpoint regression analysis of annual ACL notifications, indicating the annual percent change (APC) and the identified trend inflection point. (**c**) Cumulative ACL notification rates across Brazilian states, stratified by geographic region. Rates were calculated using the total population (all-races category) and are expressed per 1000 inhabitants. * Indicates that the Annual Percent Change (APC) is significantly different from zero (*p* < 0.05).

The temporal distribution of ACL in Brazil between 2016 and 2025 showed variation in the annual number of reported cases (Figure 1a). Notifications increased from 13,915 cases in 2016 to 18,932 in 2017, followed by a period of relatively stable transmission with consistently high case numbers between 2018 and 2023. After 2023, a marked decline was observed, with reported cases decreasing to 10,972 in 2024 and 6220 in 2025.

Joinpoint regression analysis indicated an overall downward trend in ACL notifications during the study period (Figure 1b). Between 2016 and 2023, the annual number of reported cases decreased at an annual percent change (APC) of −2.49%, followed by a steeper decline between 2023 and 2025 (APC = −34.60%).

The spatial distribution of cumulative ACL notification rates in the overall population revealed marked geographic heterogeneity across Brazil (Figure 1c). The highest cumulative notification rates were concentrated in the North region, particularly in Acre, Amapá, Roraima, Rondônia, and Amazonas. Elevated rates were also observed in parts of the Central-West and Northeast, especially in Mato Grosso, Maranhão, and Bahia, whereas the Southeast and South regions generally showed lower cumulative notification rates.

### 3.2. Race/Color Profile of Cumulative ACL Notification Rates

Table 1 summarizes cumulative ACL notification rates according to macroregion, state, and race/color group. At the national level, the cumulative ACL notification rate for the overall population was 0.72 per 1000 inhabitants (95% CI: 0.71–0.73). The highest regional cumulative notification rates were observed in the North (3.87; 95% CI: 3.84–3.90) and Central-West (1.38; 95% CI: 1.36–1.39), whereas lower rates were recorded in the Northeast (0.65; 95% CI: 0.64–0.66), Southeast (0.23; 95% CI: 0.23–0.24), and South (0.09; 95% CI: 0.08–0.09). When stratified by race/color, cumulative notification rates varied markedly across population groups and regions. Asian populations showed particularly high state-level rates in Acre (39.40; 95% CI: 30.94–49.47), Roraima (35.71; 95% CI: 23.73–51.62), and Amapá (32.09; 95% CI: 20.56–47.74). Indigenous populations presented the highest state-level rates overall, especially in Amapá (60.44; 95% CI: 55.80–65.38), Mato Grosso (26.25; 95% CI: 24.93–27.62), Rondônia (20.49; 95% CI: 18.41–22.74), and Acre (18.48; 95% CI: 16.95–20.11). Brown populations also showed elevated rates across multiple endemic areas, with the highest values observed in Acre (12.99; 95% CI: 12.69–13.29), Amapá (9.85; 95% CI: 9.57–10.14), Roraima (5.53; 95% CI: 5.29–5.78), and Mato Grosso (4.36; 95% CI: 4.27–4.45). In contrast, White and Black populations generally presented lower cumulative notification rates, especially in the South and Southeast.

**Figure 2:**
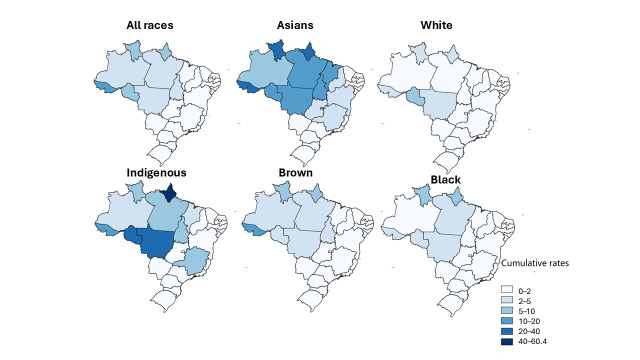
Choropleth maps showing the spatial distribution of cumulative ACL notification rates in Brazil from 2016 to 2025, stratified by race/color group. Panels display cumulative notification rates for the overall population (All races) and for Asian, White, Indigenous, Brown, and Black populations. Rates were calculated using SINAN notification data and are expressed per 1000 inhabitants. To allow direct visual comparison across panels, all maps were displayed using the same fixed class intervals in QGIS. Darker shades indicate higher cumulative notification rates.

The choropleth maps reinforced the marked geographic heterogeneity of cumulative ACL notification rates and showed distinct patterns according to race/color group (Figure 2). Overall, higher rates were concentrated mainly in the North region. The spatial distribution observed among Brown individuals closely resembled that of the overall population. In contrast, Asian and Indigenous populations showed more focal areas of high cumulative notification, whereas White and Black populations displayed more localized distributions and generally lower rates across most states.

To evaluate whether these spatial patterns represented statistically significant geographic clusters, Local Moran’s I analyses were performed (Table 2). Significant High–High (HH) clusters were concentrated predominantly in northern Brazil, especially in Acre and Rondônia, which were recurrent cluster areas across the overall population and multiple race/color groups. In the overall population and among Brown individuals, HH clusters were identified in Acre and Rondônia. Among White individuals, HH clusters were detected in Acre, Rondônia, and Mato Grosso, whereas Indigenous populations showed a more restricted pattern, with a significant HH cluster limited to Rondônia. Asian populations presented a significant HH cluster in Pará, while the broadest clustering pattern was observed among Black individuals, with HH clusters detected in Acre, Rondônia, Roraima, Pará, and Amapá.

### 3.3. Ethnic–Racial Profile of ACL Mortality

Table 3 summarizes ACL mortality rates according to macroregion, state, and race/color group. At the national level, the overall ACL mortality rate was 0.07 deaths per 100,000 inhabitants (95% CI: 0.06–0.09). The highest regional mortality rates was observed in the North (0.16; 95% CI: 0.11–0.23) and Central-West (0.14; 95% CI: 0.09–0.22), whereas lower rates was recorded in the Northeast (0.08; 95% CI: 0.06–0.11), Southeast (0.04; 95% CI: 0.03–0.11), and South (0.04; 95% CI: 0.02–0.07). When stratified by race/color at the national level, mortality estimates varied across population groups, with the highest estimate observed among Indigenous populations (0.41; 95% CI: 0.13–0.96), followed by Asian (0.12; 95% CI: 0.00–0.66), Brown (0.09; 95% CI: 0.07–0.11), Black (0.05; 95% CI: 0.02–0.09), and White populations (0.04; 95% CI: 0.03–0.06). Estimates for Indigenous and Asian populations should be interpreted cautiously because several strata were based on small numbers of deaths, resulting in wide confidence intervals.

**Figure 3:**
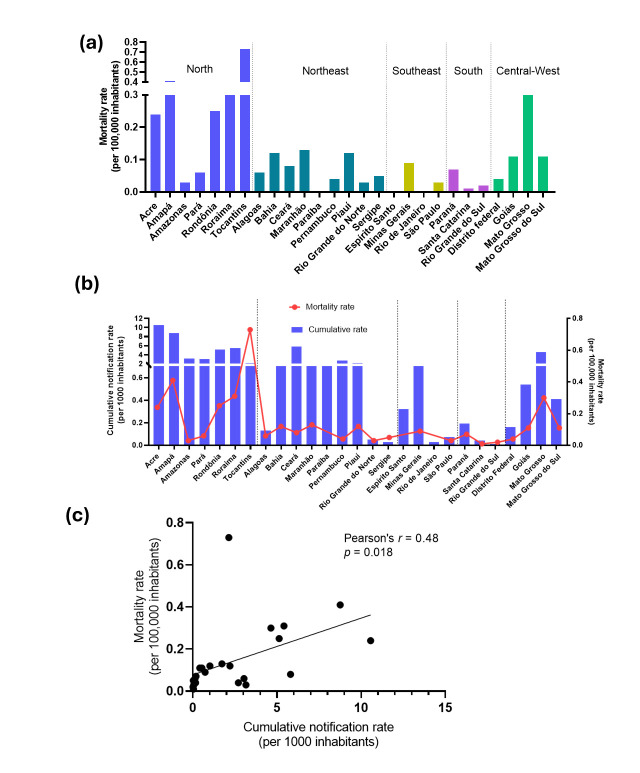
Spatial distribution and relationship between cumulative notification and mortality rates of American cutaneous leishmaniasis (ACL) in Brazil from 2016 to 2025. (**a**) State-level mortality rates (per 100,000 inhabitants), grouped according to Brazil’s geographic regions. (**b**) Comparison of cumulative notification rates (per 1000 inhabitants) and mortality rates (per 100,000 inhabitants) among Brazilian states. Rates were calculated using the total population, irrespective of race/color classification. (**c**) Pearson correlation between cumulative notification and mortality rates at the state level, demonstrating a moderate positive association (r = 0.48, *p* = 0.018).

State-level analysis showed substantial variation in ACL mortality rates across Brazil (Figure 3a). Comparison of cumulative notification and mortality rates suggested that states with higher cumulative notification rates also tended to present higher mortality rates, although with heterogeneity across states (Figure 3b). This pattern was supported by a moderate positive correlation between cumulative notification and mortality rates (Pearson’s r = 0.48; *p* = 0.018) (Figure 3c).

**Figure 4:**
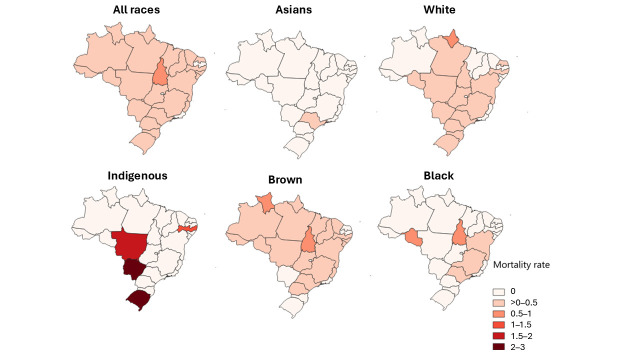
Choropleth maps showing state-level ACL mortality rates in Brazil from 2016 to 2025, stratified by race/color groups. Panels display mortality rates for the overall population (All races) and for Asian, White, Indigenous, Brown, and Black populations. Mortality rates were calculated using ACL-related deaths and population data and are expressed per 100,000 inhabitants. To allow direct visual comparison across panels, all maps were displayed using the same fixed class intervals in QGIS. Darker shades indicate higher mortality rates.

The choropleth maps reinforced the marked geographic heterogeneity of ACL mortality and showed distinct patterns according to race/color group (Figure 4). The overall population and Brown population maps showed higher mortality rates concentrated mainly in the North and Central-West regions. In contrast, the Indigenous population displayed a distinct distribution, with higher mortality rates concentrated in a limited number of states, particularly in the Central-West and South. Mortality among White, Black, and Asian populations was more localized and remained low across most states.

To evaluate whether these spatial patterns represented statistically significant geographic clusters, Local Moran’s I analyses were performed (Table 4). A significant High–High (HH) cluster was identified in Mato Grosso for the overall population (*p* = 0.048) and for White individuals (*p* = 0.033), indicating that this state and its neighboring areas shared similarly elevated mortality rates. No significant HH clusters were detected among Indigenous, Brown, Black, or Asian populations. Significant High–Low (HL) spatial outliers were identified in two population groups: Rondônia among Black individuals (*p* = 0.001) and São Paulo among Asian individuals (*p* < 0.001).

## 4. Discussion

American cutaneous leishmaniasis (ACL) remains an important public health problem in Brazil, particularly in regions characterized by intense environmental transformation and close interaction between human populations and sylvatic ecosystems [14]. In this context, understanding the temporal, spatial, and race/color-related distribution of ACL is essential to support more targeted and equitable surveillance and control strategies. The Pan American Health Organization (PAHO) recognizes ethnicity as a structural determinant of health and highlights the importance of addressing persistent social inequities through public policies [13]. Although previous studies have described the epidemiological profile of ACL in Brazil [8,15,16], analyses focusing on disparities across race/color groups remain limited. Our findings therefore contribute to a broader understanding of ACL epidemiology in Brazil by highlighting temporal trends, spatial concentration, and differences in cumulative notification and mortality patterns across race/color groups.

Our findings indicate an increase in the number of reported ACL cases between 2016 and 2017, followed by a decline from 2017 to 2025. This reduction may be partially related to the strengthening of control and surveillance activities implemented through the Tegumentary Leishmaniasis Surveillance Program (PV-LT), including expanded access to diagnosis and treatment, vector control activities, health education initiatives, and environmental surveillance measures [6]. Nevertheless, caution is warranted when interpreting the sharp reduction observed after 2023. Although data were extracted in May 2026, after the end of the 2025 surveillance year, reporting delays and the progressive consolidation of records in SINAN cannot be completely excluded. Therefore, the apparent decline observed in the final years of the series, particularly in 2025, should be interpreted with caution, as it may partially reflect delayed case reporting rather than a true epidemiological reduction. Consequently, this potential incompleteness may also have influenced the Joinpoint temporal trend analysis.

Regarding the spatial distribution of ACL, the highest cumulative notification rates were concentrated in the North region and in Mato Grosso, in the Central-West region. ACL transmission in Brazil is closely associated with sylvatic environments, phlebotomine vectors, and wild animal reservoirs [6], which may help explain the persistence of higher notification rates in these territories. The North region harbors a high diversity of reservoir hosts, including rodents, marsupials, and sloths, as well as a wide range of vector species. In addition, it presents the greatest diversity of *Leishmania* species associated with ACL, including *L. amazonensis*, *L. guyanensis*, *L. braziliensis*, *L. lainsoni*, *L. naiffi*, *L. shawi*, *L. lindenbergi*, *L. panamensis*, *L. utingensis*, and *L. mexicana* [16 17,18]. In contrast, other regions of Brazil are predominantly affected by *L. amazonensis* and *L. braziliensis* [17].

The concentration of higher cumulative notification rates in Acre, Amapá, Rondônia, Roraima, Pará, and Mato Grosso is also consistent with areas undergoing intense environmental transformation, including deforestation, mining, road construction, and agricultural expansion. Although the present study was not designed to directly evaluate the impact of these processes, previous evidence [18] suggests that such changes may increase human exposure to sylvatic transmission settings and contribute to the maintenance of ACL in these areas. This interpretation may also help explain why Mato Grosso, despite belonging to the Central-West region, presents an epidemiological profile that more closely resembles that observed in the Amazon basin.

The marked contrast between the North region and the South and Southeast regions further reinforces the environmental dependence of ACL transmission dynamics in Brazil. While the North region showed the highest cumulative notification rates, states in the South and Southeast generally exhibited substantially lower values. This distribution is consistent with the uneven geographic distribution of ecological conditions favorable to ACL transmission, including forest environments that sustain vector populations and reservoir hosts [19].

With respect to race/color distribution, Asian and Indigenous populations presented the highest cumulative notification rates in several states, particularly in the North and Central-West regions. However, rate estimates derived from relatively small population strata should be interpreted with caution. Although 95% confidence intervals were provided, limited population sizes may still contribute to statistical instability and should be considered when interpreting these findings. In contrast, elevated cumulative notification rates among Indigenous populations were observed more consistently across multiple states and regions, suggesting a more robust epidemiological pattern. The spatial distribution observed among Brown individuals closely resembled that of the overall population, indicating that the national pattern of ACL notification substantially overlaps with the distribution of cases in this group. White and Black populations generally presented lower cumulative notification rates across most states.

The elevated cumulative notification rates observed among Indigenous populations across geographically distinct states suggest that ACL burden is shaped not only by local ecological conditions, but also by broader social and territorial vulnerabilities. Indigenous communities are often disproportionately affected by territorial insecurity, environmental degradation, and barriers to healthcare access, all of which may increase exposure to transmission settings and hinder timely diagnosis and treatment [20]. In this context, the persistence of high cumulative notification rates across different regions highlights the importance of interpreting ACL not only as an environmentally determined disease, but also as a condition influenced by longstanding social inequities and unequal access to health services [17,21].

Regarding mortality, Indigenous populations showed the highest mortality rates, while Brown populations also presented elevated values in several settings. Notably, among Indigenous individuals, higher mortality rates were not restricted to the North region and were more pronounced in parts of the South and Central-West regions. This pattern may reflect delayed diagnosis, barriers to healthcare access, therapeutic challenges, or limited clinical familiarity with ACL in areas where the disease is less common [22,23]. Together, these findings suggest that the geographic distribution of mortality does not fully mirror the distribution of cumulative notification rates, indicating that the factors associated with disease occurrence and those influencing unfavorable outcomes may not be entirely the same. Mortality estimates should also be interpreted with caution, as they were derived from the outcome field available in SINAN notification records rather than from a dedicated mortality information system. Consequently, these estimates may be subject to limitations inherent to surveillance databases, including incomplete outcome registration and potential underreporting.

Our data further indicate that, although cumulative ACL notification rates were highest among Indigenous populations and, in some settings, among Asian populations, mortality disproportionately affected Indigenous populations and remained elevated among Brown populations in several states, reinforcing the existence of race/color and territorial inequalities in both disease burden and disease outcomes [24,25]. In the case of Brown populations, this pattern should be interpreted cautiously, given the broad demographic representation of this group across Brazil and its close spatial correspondence with the overall distribution of ACL notifications. Even so, the higher mortality rates observed in this group may reflect underlying social and structural vulnerabilities [12], including socioeconomic disparities that contribute to delayed diagnosis and poorer clinical outcomes. This apparent dissociation between cumulative notification and mortality suggests that the determinants of transmission and those influencing disease outcomes do not fully overlap. While cumulative notification rates are more closely related to environmental exposure and vector distribution [6], mortality is likely influenced by factors such as access to healthcare services, timeliness of diagnosis, and the quality of clinical management [22]. Regional disparities in the organization and capacity of the Unified Health System (SUS), particularly in remote and resource-limited settings, may further contribute to delays in diagnosis and treatment, thereby increasing the risk of unfavorable outcomes even in areas with only moderate cumulative notification rates [26].

In recent years, economic activities such as deforestation, mining, and the exploitation of natural resources have generated substantial environmental and social impacts that disproportionately affect Indigenous populations. Beyond territorial loss and cultural disruption, these processes may increase exposure to infectious diseases, including ACL. Factors such as inadequate sanitation, food insecurity, and frequent contact with forest environments may further contribute to the maintenance of transmission in some Indigenous territories [27]. In addition, subsistence and occupational activities, including agriculture, hunting, and fishing, may increase direct exposure to sylvatic environments, while persistent barriers to healthcare access can delay diagnosis and treatment [26,28]. Collectively, these factors may help explain the high cumulative notification and mortality rates observed among Indigenous populations and reinforce the need for targeted strategies aimed at reducing inequities in prevention, diagnosis, and care.

One of the most relevant findings of this study is the identification of substantial disparities in ACL occurrence and outcomes across race/color groups in Brazil. The disproportionate burden observed among Indigenous populations reinforces evidence that neglected tropical diseases are closely linked to social exclusion, environmental injustice, and unequal access to healthcare services. From a public health perspective, surveillance and control strategies should incorporate culturally appropriate approaches and prioritize territories where Indigenous populations face overlapping environmental and social vulnerabilities [29].

Despite the patterns identified in this study, caution is warranted when interpreting comparisons across race/color groups because race/color information was missing for a non-negligible proportion of cases in several states. For cumulative notification data, missing information was particularly high in Rio Grande do Norte (14.48%) and the Federal District (11.32%), while for mortality data, higher levels of incompleteness were observed in Bahia (17.64%), Ceará (14.28%), Maranhão (11.11%), and Paraná (12.50%). This degree of missingness may affect the accuracy of cumulative notification and mortality estimates across race/color groups and constitutes a potential source of bias that should be considered when interpreting the observed disparities. In addition, the quality of race/color completion in surveillance systems directly affects the ability to monitor health inequities and to formulate more equitable public health responses.

Taken together, these findings indicate that the epidemiological dynamics of ACL in Brazil are shaped by a complex interplay of environmental exposure, social vulnerability, and unequal access to healthcare services. The disparities observed across race/color groups suggest that the burden of ACL extends beyond transmission patterns alone, reflecting broader structural inequalities that influence both disease occurrence and clinical outcomes. These results underscore the importance of incorporating equity-oriented approaches into surveillance, prevention, and control strategies, particularly for populations disproportionately affected by the disease.

## 5. Conclusions

In conclusion, ACL in Brazil is marked by pronounced race/color and territorial inequalities, with distinct patterns of cumulative notification and mortality across population groups and regions. Indigenous populations consistently bore the greatest burden of disease, while mortality remained elevated among Indigenous populations and, in several settings, among Brown populations, underscoring that the determinants of transmission and those influencing unfavorable outcomes do not fully overlap. The concentration of cases and spatial clusters in the North and Amazon-related territories further highlights the combined role of environmental exposure, social vulnerability, and unequal access to healthcare in shaping ACL burden in the country. Together, these findings reinforce the need for surveillance, prevention, and control strategies that are not only geographically targeted, but also explicitly equity-oriented, with particular attention to historically underserved populations and territories.

## Figures and Tables

**Table 1:** Cumulative ACL notification rates and corresponding 95% confidence intervals in Brazil, stratified by official race/color categories, macroregion, and state.

American Cutaneous Leishmaniasis								
Region	State	Cumulative Rate						Unknown (%)
		All Races	Asian	White	Indigenous	Brown	Black	
North	Acre	10.56 (10.34–1.78)	39.40 (30.94–49.47)	4.07 (3.78–4.37)	18.48 (16.95–20.11)	12.99 (12.69–13.29)	3.53 (3.11–4.00)	1.19
	Amapá	8.75 (8.54–8.97)	32.09 (20.56–47.74)	2.69 (2.44–2.96)	60.44 (55.80–65.38)	9.85 (9.57–10.14)	5.94 (5.44–6.48)	3.22
	Amazonas	3.15 (3.10–3.21)	6.37 (4.51–8.75)	1.06 (0.99–1.14)	2.92 (2.73–3.12)	3.57 (3.50–3.64)	1.93 (1.74–2.13)	5.73
	Pará	3.04 (3.00–3.08)	15.69 (13.56–18.05)	1.79 (1.72–1.86)	6.75 (6.15–7.39)	3.33 (3.29–3.38)	2.50 (2.39–2.61)	2.04
	Rondônia	5.13 (5.02–5.25)	14.80 (11.37–18.93)	5.52 (5.31–5.73)	20.49 (18.41–22.74)	4.62 (4.48–4.76)	4.11 (3.78–4.46)	2.31
	Roraima	5.41 (5.23–5.60)	35.71 (23.73–51.62)	2.83 (2.55–3.14)	6.04 (5.54–6.57)	5.53 (5.29–5.78)	6.04 (5.37–6.76)	5.55
	Tocantins	2.14 (2.07–2.21)	14.10 (10.39–18.69)	1.31 (1.19–1.43)	9.29 (7.96–10.77)	2.40 (2.30–2.50)	1.46 (1.30–1.64)	2.24
	Total	3.87 (3.84–3.90)	19.75 (18.18–21.42)	2.03 (1.98–2.08)	5.91 (5.71–6.12)	4.64 (4.60–4.68)	2.92 (2.84–3.01)	2.94
Northeast	Alagoas	0.13 (0.12–0.0.15)	0.36 (0.04–1.31)	0.07 (0.06–0.09)	0.55 (0.27–0.98)	0.16 (0.14–0.18)	0.12 (0.08–0.16)	2.40
	Bahia	1.01 (0.99–1.02)	4.43 (3.46–5.59)	0.35 (0.33–0.38)	1.63 (1.36–1.92)	1.21 (1.19–1.24)	0.82 (0.79–0.85)	5.14
	Ceará	0.58 (0.57–0.60)	1.51 (0.88–2.42)	0.30 (0.28–0.32)	0.75 (0.51–1.07)	0.72 (0.70–0.75)	0.30 (0.25–0.34)	1.37
	Maranhão	1.73 (1.70–1.77)	13.30 (10.65–16.41)	0.98 (0.93–1.03)	3.31 (2.85–3.83)	1.99 (1.95–2.03)	1.37 (1.29–1.45)	1.55
	Paraíba	0.15 (0.14–0.17)	1.02 (0.33–2.38)	0.06 (0.05–0.08)	1.45 (1.02–2.00)	0.18 (0.16–0.20)	0.10 (0.07–0.15)	9.09
	Pernambuco	0.27 (0.26–0.28)	1.13 (0.63–1.87)	0.14 (0.13–0.16)	0.22 (0.13–0.34)	0.33 (0.31–0.34)	0.19 (0.16–0.22)	7.14
	Piauí	0.22 (0.20–0.23)	2.27 (0.91–4.69)	0.20 (0.17–0.23)	0.32 (0.04–1.17)	0.21 (0.20–0.24)	0.16 (0.13–0.21)	6.11
	Rio Grande do Norte	0.05 (0.04–0.06)	0.19 (0.00–1.06)	0.04 (0.03–0.05)	0.11 (0.00–0.59	0.05 (0.04–0.06)	0.03 (0.02–0.06)	14.48
	Sergipe	0.03 (0.02–0.04)	0.67 (0.08–2.43)	0.02 (0.01–0.03)	0.22 (0.01–1.22)	0.03 (0.02–0.04)	0.03 (0.01–0.06)	6.25
	Total	0.65 (0.64–0.66)	6.84 (6.23–7.48)	0.26 (0.26–0.27)	1.27 (1.15–1.40)	0.79 (0.78–0.80)	0.60 (0.58–0.61)	3.66
Southeast	Espírito Santo	0.32 (0.30–0.33)	14.76 (11.34–18.89)	0.47 (0.43–0.51)	0.26 (0.05–0.75)	0.28 (0.26–0.30)	0.31 (0.26–0.37)	4.16
	Minas Gerais	0.73 (0.72–0.74)	3.19 (2.60–3.87)	0.49 (0.48–0.51)	7.25 (6.34–8.24)	0.88 (0.86–0.90)	0.56 (0.53–0.59)	5.68
	Rio de Janeiro	0.03 (0.03–0.04)	0.23 (0.07–0.53)	0.04 (0.03–0.04)	0.13 (0.02–0.45)	0.02 (0.02–0.03)	0.02 (0.02–0.03)	12.41
	São Paulo	0.07 (0.06–0.07)	0.07 (0.05–0.09)	0.07 (0.07–0.08)	0.46 (0.29–0.68)	0.05 (0.05–0.06)	0.05 (0.05–0.06)	4.75
	Total	0.23 (0.23–0.24)	0.40 (0.35–0.45)	0.19 (0.19–0.20)	2.67 (2.38–3.00)	0.32 (0.32–0.33)	0.21 (0.20–0.22)	5.61
South	Paraná	0.19 (0.18–0.20)	0.24 (0.15–0.36)	0.22 (0.21–0.23)	0.29 (0.12–0.56)	0.12 (0.11–0.13)	0.16 (0.13–0.20)	1.86
	Santa Catarina	0.04 (0.03–0.04)	0.16 (0.02–0.58)	0.04 (0.04–0.05)	0.00 (0.00–0.19)	0.03 (0.02–0.04)	0.06 (0.04–0.10)	1.02
	Rio Grande do Sul	0.01 (0.01–0.02)	0.00 (0.00–0.45)	0.01 (0.01–0.02)	0.03 (0.00–0.16)	0.01 (0.00–0.02)	0.02 (0.01–0.03)	5.00
	Total	0.09 (0.08–0.09)	0.22 (0.15–0.33)	0.12 (0.12–0.13)	0.20 (0.11–0.32)	0.09 (0.08–0.10)	0.10 (0.08–0.12)	1.93
Central-West	Distrito Federal	0.16 (0.15–0.18)	0.11 (0.10–0.13)	0.16 (0.02–0.56)	0.12 (0.10–0.15)	0.90 (0.29–2.11)	0.10 (0.07–0.14)	11.32
	Goiás	0.54 (0.53–0.56)	3.36 (2.54–4.35)	0.44 (0.42–0.47)	1.05 (0.53–1.89)	0.56 (0.54–0.58)	0.60 (0.54–0.66)	3.70
	Mato Grosso	4.64 (4.57–4.71)	10.54 (8.70–12.67)	4.31 (4.19–4.43)	26.25 (24.93–27.62)	4.36 (4.27–4.45)	3.09 (2.91–3.27)	2.09
	Mato Grosso do Sul	0.41 (0.39–0.43)	0.92 (0.54–1.45)	0.36 (0.33–0.40)	0.73 (0.57–0.92)	0.39 (0.35–0.42)	0.36 (0.28–0.46)	5.83
	Total	1.38 (1.36–1.39)	4.63 (4.10–5.21)	1.52 (1.49–1.55)	11.78 (11.27–12.31)	1.74 (1.71–1.77)	1.48 (1.42–1.55)	2.73
Brazil	Total	0.72 (0.71–0.73)	0.01 (0.00–0.01)	0.13 (0.11–0.14)	0.03 (0.03–0.04)	0.47 (0.45–0.49)	0.06 (0.05–0.04)	3.26

ACL = American Cutaneous Leishmaniasis; 95% CI = 95% confidence interval; Unknown (%) = proportion of reported cases with missing race/color information.

**Table 2:** States presenting statistically significant High–High (HH) clusters identified by Local Moran’s I analysis of cumulative ACL notification rates according to ethnic–racial group (*p* < 0.05).

Population Group	Significant HH * Clusters
All races	Acre, Rondônia
Brown	Acre, Rondônia
White	Acre, Rondônia, Mato Grosso
Indigenous	Rondônia
Asian	Pará
Black	Acre, Rondônia, Roraima, Pará, Amapá

* HH = High–High cluster identified by Local Moran’s I analysis.

**Table 3:** ACL mortality rates in Brazil, stratified by ethnic–racial groups, macro-region and state.

American Cutaneous Leishmaniasis								
Region	State	Mortality Rate (95% CI *)						Unknown (%)
		All Races	Asian	White	Indigenous	Brown	Black	
North	Acre	0.24 (0.03–0.87)	0.00 (0.00–196.43)	0.00 (0.00–2.07)	0.00 (0.00–12.65)	0.36 (0.04–1.31)	0.00 (0.00–5.19)	0.00
	Amapá	0.41 (0.08–1.19)	0.00 (0.00–493.17)	0.64 (0.02–3.55)	0.00 (0.00–35.68)	0.42 (0.05–1.51)	0.00 (0.00–4.26)	0.00
	Amazonas	0.03 (0.00–0.14)	0.00 (0.00–61.86)	0.00 (0.00–0.51)	0.00 (0.00–1.21)	0.04 (0.00–0.21)	0.00 (0.00–1.90)	0.00
	Pará	0.06 (0.02–0.14)	0.00 (0.00–29.67)	0.13 (0.02–0.46)	0.00 (0.00–5.33)	0.05 (0.01–0.15)	0.00 (0.00–0.46)	0.00
	Rondônia	0.25 (0.07–0.65)	0.00 (0.00–86.65)	0.21 (0.01–1.15)	0.00 (0.00–21.35)	0.21 (0.03–0.77)	0.73 (0.02–4.07)	0.00
	Roraima	0.31 (0.04–1.13)	0.00 (0.00–470.52)	0.00 (0.00–2.81)	0.00 (0.00–4.10)	0.55 (0.07–1.98)	0.00 (0.00–7.50)	0.00
	Tocantins	0.73 (0.36–1.30)	0.00 (0.00–108.34)	0.29 (0.01–1.59)	0.00 (0.00–19.69)	0.96 (0.44–1.82)	0.50 (0.01–2.79)	0.00
	Total	0.16 (0.11–0.23)	0.00 (0.00–12.52)	0.14 (0.05–0.32)	0.00 (0.00–0.68)	0.18 (0.11–0.27)	0.13 (0.02–0.47)	0.00
Northeast	Alagoas	0.06 (0.01–0.23)	0.00 (0.00–67.01)	0.00 (0.00–0.40)	0.00 (0.00–18.36)	0.11 (0.01–0.38)	0.00 (0.00–1.23)	0.00
	Bahia	0.12 (0.07–0.19)	0.00 (0.00–23.03)	0.04 (0.00–0.20)	0.00 (0.00–4.41)	0.10 (0.04–0.19)	0.16 (0.05–0.37)	17.64
	Ceará	0.08 (0.03–0.16)	0.00 (0.00–32.77)	0.00 (0.00–0.15)	0.00 (0.00–9.23)	0.11 (0.04–0.23)	0.00 (0.00–0.62)	14.28
	Maranhão	0.13 (0.06–0.25)	0.00 (0.00–56.40)	0.00 (0.00–0.27)	0.00 (0.00–6.75)	0.18 (0.08–0.35)	0.00 (0.00–0.43)	11.11
	Paraíba	0.00 (0.00–0.09)	0.00 (0.00–75.10)	0.00 (0.00–0.26)	0.00 (0.00–14.48)	0.00 (0.00–0.17)	0.00 (0.00–1.17)	0.00
	Pernambuco	0.04 (0.01–0.11)	0.00 (0.00–27.89)	0.03 (0.00–0.18)	1.20 (0.03–6.66)	0.04 (0.00–0.14)	0.00 (0.00–0.41)	0.00
	Piauí	0.12 (0.03–0.31)	0.00 (0.00–119.85)	0.00 (0.00–0.50)	0.00 (0.00–59.52)	0.19 (0.05–0.48)	0.00 (0.00–0.92)	0.00
	Rio Grande do Norte	0.03 (0.00–0.17)	0.00 (0.00–70.44)	0.08 (0.00–0.43)	0.00 (0.00–39.31)	0.00 (0.00–0.22)	0.00 (0.00–1.22)	0.00
	Sergipe	0.05 (0.00–0.25)	0.00 (0.00–123.87)	0.00 (0.00–0.66)	0.00 (0.00–80.54)	0.07 (0.00–0.41)	0.00 (0.00–1.30)	0.00
	Total	0.08 (0.06–0.11)	0.00 (0.00–5.37)	0.02 (0.00–0.06)	0.31 (0.01–1.70)	0.10 (0.07–0.14)	0.07 (0.02–0.16)	11.11
Southeast	Espírito Santo	0.00 (0.00–0.10)	0.00 (0.00–86.43)	0.00 (0.00–0.25)	0.00 (0.00–31.75)	0.00 (0.00–0.19)	0.00 (0.00–0.86)	0.00
	Minas Gerais	0.09 (0.06–0.14)	0.00 (0.00–11.64)	0.08 (0.03–0.17)	0.00 (0.00–11.57)	0.09 (0.04–0.18)	0.08 (0.01–0.30)	5.26
	Rio de Janeiro	0.00 (0.00–0.02)	0.00 (0.00–16.89)	0.00 (0.00–0.05)	0.00 (0.00–23.19)	0.00 (0.00–0.06)	0.00 (0.00–0.14)	0.00
	São Paulo	0.03 (0.02–0.06)	0.19 (0.00–1.09)	0.03 (0.01–0.06)	0.00 (0.00–7.30)	0.03 (0.01–0.08)	0.06 (0.01–0.20)	0.00
	Total	0.04 (0.03–0.11)	0.00 (0.00–0.65)	0.03 (0.02–0.06)	0.00 (0.00–3.36)	0.04 (0.02–0.07)	0.04 (0.01–0.10)	2.94
South	Paraná	0.07 (0.03–0.14)	0.00 (0.00–3.68)	0.07 (0.02–0.16)	0.00 (0.00–13.17)	0.06 (0.01–0.21)	0.00 (0.00–0.76)	12.5
	Santa Catarina	0.01 (0.00–0.07)	0.00 (0.00–29.66)	0.00 (0.00–0.10)	0.00 (0.00–19.12)	0.00 (0.00–0.25)	0.00 (0.00–1.19)	0.00
	Rio Grande do Sul	0.02 (0.00–0.07)	0.00 (0.00–45.22)	0.01 (0.00–0.01)	2.93 (0.07–16.30)	0.00 (0.00–0.23)	0.00 (0.00–0.52)	0.00
	Total	0.04 (0.02–0.07)	0.00 (0.00–3.05)	0.03 (0.01–0.07)	1.23 (0.03–6.86)	0.03 (0.00–0.11)	0.00 (0.00–0.25)	0.00
Central-West	Federal District	0.04 (0.00–0.20)	0.00 (0.00–28.80)	0.00 (0.00–0.33)	0.00 (0.00–66.63)	0.07 (0.00–0.41)	0.00 (0.00–1.22)	0.00
	Goiás	0.11 (0.05–0.22)	0.00 (0.00–21.72)	0.04 (0.00–0.22)	0.00 (0.00–35.36)	0.18 (0.07–0.38)	0.00 (0.00–0.57)	0.00
	Mato Grosso	0.30 (0.15–0.54)	0.00 (0.00–34.12)	0.34 (0.09–0.87)	1.76 (0.04–9.83)	0.29 (0.11–0.64)	0.00 (0.00–1.02)	0.00
	Mato Grosso do Sul	0.11 (0.02–0.32)	0.00 (0.00–18.81)	0.09 (0.00–0.48)	2.08 (0.25–7.52)	0.00 (0.00–0.29)	0.00 (0.00–2.06)	0.00
	Total	0.14 (0.09–0.22)	0.00 (0.00–6.12)	0.10 (0.04–0.22)	1.78 (0.37–5.21)	0.16 (0.09–0.27)	0.00 (0.00–0.25)	0.00
Brazil		0.07 (0.06–0.09)	0.12 (0.00–0.66)	0.04 (0.03–0.06)	0.41 (0.13–0.96)	0.09 (0.07–0.11)	0.05 (0.02–0.09)	4.34

* 95% CI = 95% confidence interval; Unknown (%) = proportion of reported cases with missing race/color information.

**Table 4:** States presenting statistically significant spatial patterns identified by Local Moran’s I analysis of cumulative ACL mortality rates according to ethnic–racial groups. Only statistically significant results (*p* < 0.05) are presented.

Population Group	Pattern	State
All races	HH	Mato Grosso
Brown	-	-
Indigenous	-	-
White	HH	Mato Grosso
Asian	HL	São Paulo
Black	HL	Rondônia

HH = High–High clusters (states with high mortality rates surrounded by neighboring states with similarly high rates); HL = High–Low spatial outliers (states with high mortality rates surrounded by neighboring states with low rates).

## Data Availability

The datasets analyzed in this study are publicly available through official Brazilian government databases. Epidemiological data were obtained from the Information System for Notifiable Diseases (SINAN), accessed via the Brazilian Ministry of Health platform (https://datasus.saude.gov.br/acesso-a-informacao/doencas-e-agravos-de-notificacao-de-2007-em-diante-sinan/accessed on 12 March 2026). Population data were retrieved from the 2022 Demographic Census conducted by the Brazilian Institute of Geography and Statistics (IBGE) (https://www.ibge.gov.br/estatisticas/sociais/trabalho/22827-censo-demografico-2022.html, accessed on 12 March 2026). Additional information may be obtained from the corresponding author upon reasonable request.

## Abbreviations

ACL	American Cutaneous Leishmaniasis
WHO	World Health Organization
PAHO	Pan American Health Organization
SINAN	Notifiable Diseases Information System
DATASUS	Department of Informatics of the Brazilian Unified Health System
IBGE	Brazilian Institute of Geography and Statistics
SUS	Sistema Único de Saúde (Brazilian Unified Health System)
QGIS	Quantum Geographic Information System
